# Skin Cancer Knowledge, Sun Exposure, Photoprotection Behavior, and Perceived Barriers Associated with Skin Cancer Types in a Greek Cohort: A Cross-Sectional Study on the Island of Crete

**DOI:** 10.3390/cancers16244226

**Published:** 2024-12-18

**Authors:** Dimitra Koumaki, Georgios Evangelou, Stamatios Gregoriou, Stamatoula Kouloumvakou, Andreas Manios, Alexander Katoulis, Georgios Vasileiou Zacharopoulos, Pavel Viktorovich Chernyshov, Marios Papadakis, Dimitrios Kassotakis, Georgios A. Manios, Evangelia Rovithi, Kyriaki Zografaki, Aikaterini Doxastaki, Ioanna Gkiaouraki, Danae Petrou, Faidra Marazaki, Dimitrios Mylonakis, Eelco de Bree, Konstantinos Krasagakis

**Affiliations:** 1 Dermatology Department, University Hospital of Heraklion, 71110 Heraklion, Greece; gevangelou@pagni.gr (G.E.); eva.rovithi@pagni.gr (E.R.); kikizog@yahoo.gr (K.Z.); katerina.doxastaki@gmail.com (A.D.); medp2012101@med.uoc.gr (I.G.); dpetrou@pagni.gr (D.P.); fmarazaki@pagni.gr (F.M.); mylonis@hotmail.com (D.M.); krasagak@uoc.gr (K.K.); 2 1st Department of Dermatology and Venereology, Andreas Sygros Hospital, National and Kapodistrian University of Athens, Medical School of Athens, I. Dragoumi 5, 16121 Athens, Greece; stamgreg@med.uoa.gr; 3 2nd Department of Internal Medicine, Sismanoglio General Hospital, Sismanogliou 37, 15126 Marousi, Greece; skoulou@auth.gr; 4 Plastic Surgery Unit, Department of Surgical Oncology, University Hospital of Heraklion, 71110 Heraklion, Greece; agmanios@pagni.gr (A.M.); gzachar@pagni.gr (G.V.Z.); dkassotakis@pagni.gr (D.K.); 5 2nd Department of Dermatology and Venereology, National and Kapodistrian University of Athens, Medical School, “Attikon” General University Hospital, Rimini 1, 12462 Haidari, Greece; akatoulis@med.uoa.gr; 6 Department of Dermatology and Venereology, National Medical University, 01601 Kyiv, Ukraine; chernyshovpavel@ukr.net; 7 Department of Surgery, Helios Clinic, University Hospital Witten-Herdecke, Heusnerstr 40, 42283 Wuppertal, Germany; mpapadakis@med.uoc.gr; 8 Department of Computer Science and Biomedical Informatics, University of Thessaly, 35131 Lamia, Greece; gmanios@uth.gr; 9 Department of Surgical Oncology, University Hospital of Heraklion, 71110 Heraklion, Greece; debree@med.uoc.gr

**Keywords:** photoprotection, basal cell carcinoma, squamous cell carcinoma, malignant melanoma, sunscreen, skin cancer

## Abstract

Background: This study aimed to explore the relationship between different types of skin cancer and factors such as sun exposure and photoprotection measures in a Greek cohort on the island of Crete. Methods: This cross-sectional observational study was conducted in the Dermatology Department of the University Hospital in Heraklion, Crete, between January 2019 and January 2024. The study population included consecutive patients diagnosed with basal cell carcinoma (BCC), squamous cell carcinoma (SCC), and malignant melanoma (MM), as well as healthy controls. The participants completed a self-reported questionnaire covering demographic and clinical data as well as questions about sun exposure and photoprotection habits. Results: A total of 265 skin cancer patients and 106 healthy controls participated in the study: 50.6% of the patients had basal cell carcinoma, 35.1% had squamous cell carcinoma, and 14.3% had malignant melanoma. The cohort comprised 41.5% females and 58.5% males, with a mean age of 73.2 years. As expected, the patients with skin cancer had a lighter skin phototype compared to the healthy controls (*p* < 0.01). They also reported greater occupational (*p* < 0.01) and leisure sun exposure (*p* < 0.01) and a higher median number of vacation weeks spent outdoors before the age of 18 (*p* = 0.030). Furthermore, the healthy controls were more likely to use sunscreen (*p* = 0.035) and preferred higher SPF (>50) when they did so (*p* < 0.01). The healthy controls also reported more frequent use of sunglasses (*p* < 0.01), brimmed hats (*p* < 0.01), and long-sleeved clothing (*p* < 0.01) compared to the skin cancer patients. Conclusion: This is the first study to analyze sun exposure and photoprotection behaviors in patients with nonmelanoma skin cancer (NMSC) and malignant melanoma (MM) in Crete, revealing the key associations and underscoring the need for targeted prevention strategies.

## Simple Summary

This study examined the link between skin cancer types and sun exposure or photoprotection habits in a Greek cohort on the island of Crete. It included 265 skin cancer patients (BCC, SCC, MM) and 106 healthy controls. The patients with skin cancer had lighter skin phototypes, higher sun exposure (occupational, leisure, and during childhood), and fewer photoprotection habits. The healthy controls used sunscreen more frequently, preferred SPF > 50, and were more likely to wear sunglasses, brimmed hats, and long-sleeved clothing. These findings highlight the need for targeted prevention strategies to reduce skin cancer risk by improving photoprotection practices, particularly in sun-exposed populations.

## 1. Introduction

Skin cancer is the most common cancer globally, with the rising incidence rates primarily linked to ultraviolet (UV) radiation exposure [1]. In 2012, the American Cancer Society estimated that 3.3 million people were diagnosed with 5.4 million cases of nonmelanoma skin cancers (NMSCs), with nearly 80% being basal cell carcinoma (BCC) [1,2,3,4]. A 2006 survey reported 3.5 million treated NMSCs in the U.S. [4,5,6,7,8]. Geographic variation shows the highest incidence in Australia and New Zealand (42 and 31 cases per 100,000 person-years for males and females), followed by Western Europe (19 cases), North America (18 cases), and Northern Europe (17–18 cases) [9]. The melanoma risk correlates with the lower latitude and higher UV indices in non-Hispanic Whites, but no evidence links UV exposure to the melanoma prevalence in Black or Hispanic populations [10,11,12,13,14]. NMSCs are associated with cumulative sun exposure and commonly occur in sun-exposed areas like the face, hands, and forearms [15]. Melanomas, however, often develop in less exposed areas, such as the legs in females and the back in males, and are linked to intense, intermittent sun exposure and sunburns [16,17,18,19]. UV radiation spans 100–400 nm, divided into UVA (315–400 nm), UVB (280–315 nm), and UVC (100–280 nm). UVB exposure is more strongly associated with melanoma than UVA.

Heraklion, Crete, has a Mediterranean climate with seasonal UV index fluctuations: 2–4 in winter, peaking at 11 in summer, and decreasing in autumn. The sunshine hours range from 5 to 12 daily, depending on the season. The higher melanoma rates in the equatorial regions align with intense UVB radiation, while UVA shows less variation across latitudes. Clinical studies link cumulative solar exposure and sunburn frequency to skin damage and NMSC risk [18]. The recommended preventive measures include physical barriers (e.g., sunglasses, hats, clothing), sun avoidance during peak hours, and sunscreen use [18,19,20,21,22,23,24,25,26]. Organizations like the American Cancer Society and Cancer Council Australia endorse these strategies, but adherence remains low, contributing to the rising skin cancer rates [27,28].

This study examines the relationship between skin cancer, sun exposure, and photoprotection measures within a Greek cohort on Crete. Mediterranean populations with Fitzpatrick skin types III–IV have moderate melanin protection but remain vulnerable to UV damage during high-exposure periods. This olive or tan skin type tans easily and rarely burns, reducing the sunburn risk but posing long-term risks like hyperpigmentation. By analyzing demographic and clinical profiles and sun safety practices, this research aims to identify the educational gaps and barriers to effective photoprotection, aiding the development of targeted interventions to reduce skin cancer incidence.

## 2. Materials and Methods

### 2.1. Study Population and Design

Between January 2019 and January 2024, the Dermatology Department at the University Hospital in Heraklion, Crete, Greece, conducted a cross-sectional observational study. The study population included consecutive adult patients diagnosed within the past three months with basal cell carcinoma (BCC), squamous cell carcinoma (SCC), or malignant melanoma (MM), as well as healthy controls without skin cancer. The controls were recruited from the same geographic region in Heraklion to ensure comparability in the environmental and lifestyle factors. The recruitment occurred through general outpatient clinics.

The exclusion criteria for the control group included any prior diagnosis of skin cancer and significant sun-related dermatological conditions. The participants provided informed consent, and study participation was voluntary. Individuals were excluded if they failed to sign the informed consent form, were unable to complete the questionnaire due to educational limitations, were under 18 years of age, or had been diagnosed with skin cancer more than three months before the study’s start.

The participants completed a self-administered questionnaire, written in Greek, which collected sociodemographic data, medical history, clinical details (e.g., Fitzpatrick skin type), sun exposure and sunburn history, lifestyle factors, and photoprotection behaviors. The questionnaire was tested and validated on a subgroup of participants prior to its full deployment.

### 2.2. Questionnaire

The survey assessed skin cancer risk factors, including the skin phototype, the sunburn history, intentional tanning, the type of skin cancer, the medical history, medications, comorbidities, habits, and the use of sunbeds or phototherapy. It also gathered information on lifestyle factors and personal or family history of skin cancers. The skin phototypes were classified using the Fitzpatrick scale (I–VI) [29].

To evaluate photoprotection knowledge, a modified questionnaire based on the International Transplant Skin Cancer Collaborative (ITSCC) program was used. The questions on photoprotective behaviors were adapted from ITSCC resources and materials provided by the Public Health Agency of Canada’s National Workshop on Measurement of Sun-Related Behaviors. These included queries about the use of broad-brimmed hats, sunscreen, protective clothing, and avoidance of sun exposure during peak UV radiation (UVR) hours.

The survey also explored photoprotective education by asking participants which healthcare provider delivered this information, when it was provided, and their preferred mode of education. Additionally, the participants identified the obstacles preventing them from adopting photoprotective measures.

### 2.3. Statistical Analysis

A descriptive statistical analysis was conducted for all of the variables. The data related to survey questions and demographics were presented using percentages and frequencies. The continuous variables were reported as the number of valid cases, mean, and standard deviation (SD), based on the results of the Kolmogorov–Smirnov test. The categorical variables were expressed as both absolute and relative frequencies for each category, relative to the total number of valid cases (n). Pearson’s chi-square test or Fisher’s exact test was used to evaluate the relationships between the categorical variables, while ANOVA and Kruskal–Wallis one-way ANOVA were applied to compare the categorical and continuous variables. Logistic regression analysis was performed to identify the independent predictors of skin cancer in the cohort. To account for the multiple comparisons and control the family-wise error rate, the Bonferroni correction was applied. This method adjusts the significance threshold (α) by dividing the conventional level of significance (0.05) by the total number of comparisons performed. The use of this correction ensures the robustness of the statistical analyses and minimizes the likelihood of type I errors. All of the *p*-values were evaluated against the adjusted threshold to determine statistical significance. The statistical significance was set at *p* < 0.05. All of the analyses were conducted using SPSS IBM 24.

### 2.4. Ethical Considerations

This observational study received approval from the Institutional Ethics Committee of the University Hospital of Heraklion, Crete, Greece (protocol code 5767, approved on 13 April 2022). Written informed consent was obtained from all of the participants before their enrollment in the study.

## 3. Results

### 3.1. Demographic and Clinical Profiles of the Study Population

A total of 265 patients with skin cancer and 106 healthy controls without any past medical history (PMH) of skin cancer, amounting to 371 individuals, were included in the study. Among the 265 skin cancer patients, 50.6% (134/265) had basal cell carcinoma, 35.1% (93/265) had squamous cell carcinoma, and 14.3% (38/265) had malignant melanoma.

The skin cancer patient population consisted of 41.9% (111/265) females and 58.1% (154/265) males, with a mean age of 72.18 ± 12.08 years. In the healthy control group, 40.6% (43/106) were females and 59.4% (63/106) were males. The patients with malignant melanoma were notably younger, with a mean age of 57.45 ± 10.75 years, compared to those with basal cell carcinoma and squamous cell carcinoma, who had mean ages of 73.58 ± 11.10 years and 76.19 ± 9.25 years, respectively.

As expected, the patients with skin cancer had a lighter skin phototype compared to the healthy controls (*p* < 0.01). They also reported higher levels of occupational (*p* < 0.01) and leisure sun exposure (*p* < 0.01), as well as a greater median number of vacation weeks spent outdoors before the age of 18 (*p* = 0.030). Table 1 provides a summary of the characteristics of the entire study population.

### 3.2. Sun Protection Practices of the Participants

The participants’ photoprotective behavioral patterns are summarized in Table 2. The survey showed that while approximately half of the patients with skin cancer, 50.6% (134/265), used sunscreen, a lower proportion was observed among the patients with squamous cell carcinoma (SCC), at 43% (40/93), compared to 50.7% (68/134) of the basal cell carcinoma (BCC) patients and 68.4% (26/38) of the malignant melanoma (MM) patients. The patients with skin cancer used sunscreen less frequently (*p* = 0.35) and the sunscreen had a lower SPF rating (*p* < 0.01). The healthy controls used UV sunglasses (*p* < 0.01), brimmed hats (*p* = 0.01), and long sleeves (*p* = 0.01) more frequently than the skin cancer patients.

### 3.3. Education, Knowledge, and Barriers Regarding Photoprotection

Only about one-third of the patients with skin cancer, 37.3% (100/265), recalled receiving photoprotection education. Specifically, 32.5% (86/265) had received sun protection advice from a specialist doctor, while only 4.9% (13/265) had received such advice from a family doctor. Additionally, 19.2% (51/265) reported receiving sun protection information from the media. The majority of the participants, 70.9% (188/265), expressed an interest in receiving photoprotection advice from a healthcare professional (Supplementary Table S1). The most commonly reported barrier to implementing sun-safe practices among skin cancer patients was skepticism, with 32.5% (86/265) stating, “I do not believe skin cancer is a serious health threat”. Financial concerns were cited by 28.7% (76/265), while 24.5% (65/265) reported that sunscreen was uncomfortable or unpleasant to use. Additionally, 23.4% (62/265) indicated that the hassle or a lack of time was a barrier (Supplementary Table S2).

## 4. Discussion

This study provides valuable insights into the demographic characteristics, sun exposure patterns, and photoprotective behaviors of patients with skin cancer compared to healthy controls within a cohort from the island of Crete, Greece. The findings underscore the key differences in age, gender distribution, and lifestyle factors contributing to the development of skin cancer, offering critical implications for targeted prevention strategies [15,16,17].

The distribution of skin cancer types in this study aligns with global trends, with basal cell carcinoma (BCC) being the most common (50.6%), followed by squamous cell carcinoma (SCC, 35.1%) and malignant melanoma (MM, 14.3%). Notably, the patients with MM were younger than those with BCC or SCC, suggesting distinct biological or behavioral risk factors contributing to MM onset. The predominance of males (58.1%) among the skin cancer patients is consistent with prior research, possibly reflecting greater sun exposure or lower adherence to photoprotective measures among men compared to women.

As expected, the patients with skin cancer had lighter skin phototypes compared to the healthy controls, reinforcing the well-established link between fair skin and a heightened skin cancer risk. Additionally, the patients with skin cancer reported significantly higher levels of both occupational and leisure-related sun exposure, as well as more frequent childhood vacations spent outdoors. These findings emphasize the cumulative impact of UV exposure over a lifetime, particularly during the early years, on skin cancer risk. This highlights the importance of early education on sun protection to mitigate the long-term risks.

The behavioral analysis revealed suboptimal photoprotection habits among the patients with skin cancer. Despite nearly half of the patients reporting sunscreen use, significant differences were observed among the cancer types, with MM patients using sunscreen more frequently (68.4%) than those with SCC (43%) or BCC (50.7%). This discrepancy may reflect the greater awareness among MM patients, possibly due to its more aggressive nature and related public health messaging. However, the sunscreen used by the patients with skin cancer generally had a lower SPF compared to that used by the healthy controls, indicating gaps in effective photoprotection practices.

The healthy controls demonstrated superior adherence to other photoprotective measures, such as wearing UV-protective sunglasses, broad-brimmed hats, and long-sleeved clothing. These findings highlight the importance of promoting comprehensive photoprotection strategies beyond sunscreen use, especially among high-risk populations. Public health campaigns should emphasize the synergistic benefits of combining multiple protective measures to maximize the UV protection.

Overall, this study reveals significant disparities in sun exposure and photoprotection habits between patients with skin cancer and healthy individuals. These findings emphasize the need for targeted interventions focusing on high-risk groups, particularly those with fair skin, high UV exposure, and inadequate photoprotection practices. Prevention programs should prioritize education on effective sunscreen use, encourage protective clothing, and target younger populations to instill lifelong sun-safe behaviors.

The relationship between sun exposure and skin cancer development is well-established, with UV radiation recognized as a significant risk factor for both nonmelanoma (BCC and SCC) and melanoma skin cancers [15,16,17,18]. Previous studies have demonstrated that photoprotection measures, including sunscreen use, protective clothing, and seeking shade, effectively reduce the skin cancer risk [19,20,21,22,23]. Despite these benefits, adherence to photoprotection practices remains suboptimal, particularly among high-risk populations such as individuals with a history of skin cancer [24].

The current photoprotection guidelines recommend the daily, year-round use of broad-spectrum sunscreen (protecting against both UVA and UVB rays) with a minimum SPF of 30. Sunscreen should be applied 30 min before UV exposure, with more frequent reapplication during activities involving heavy sweating, swimming, or towel drying [28]. In addition to sunscreen, the use of UV-filtering sunglasses, broad-brimmed hats, and tightly woven long-sleeved shirts and trousers is strongly encouraged [29,30,31].

Several studies have examined the relationship between sun exposure, photoprotection behaviors, and perceived barriers to sun safety, similar to our findings in Crete [32,33,34,35,36,37,38,39,40,41,42,43,44,45,46]. One national database study showed that sunscreen use, seeking shade, and wearing protective clothing were associated with a lower risk of developing skin cancer [47]. However, the study also noted that wearing long-sleeved shirts did not consistently show a protective effect, emphasizing the need to understand the demographic factors influencing these behaviors.

Research conducted in Atlantic Canada revealed a “sunscreen paradox”, where the participants acknowledged the risks of sun exposure but showed inconsistent sun protection behaviors. This highlights the need for improved public health messaging and education on effective strategies to reduce melanoma incidence [48]. A multicenter case–control study in Spain compared sun exposure habits and photoprotection measures among patients with BCC, SCC, and melanoma against a control group. The patients with skin cancer reported higher past sun exposure but also utilized photoprotection measures such as avoiding peak sun hours and using sunscreen. However, the melanoma patients were less likely to use clothing and seek shade compared to the BCC and SCC patients, indicating differences in protective behaviors by skin cancer type [33].

The common barriers to effective sun protection identified in studies include skepticism about skin cancer seriousness, financial concerns regarding sunscreen costs, and the discomfort associated with sunscreen use. These barriers were also observed in our study, where many participants expressed doubts about the necessity of sun protection and cited practical challenges in implementing protective measures [49].

All of these studies collectively emphasize the importance of understanding the behavioral and perceptual factors influencing sun protection practices across populations [38,39,40,41,42,43,44,45,47,50,51,52,53,54,55,56,57,58]. Tailored educational interventions addressing these barriers are essential to reduce the skin cancer incidence.

Our study uniquely focuses on a Greek cohort from Crete, providing valuable insights into skin cancer within a Mediterranean context. This regional perspective allows for a better understanding of how local environmental factors, cultural practices, and genetic predispositions influence the skin cancer incidence and prevention strategies. By using a detailed questionnaire capturing sociodemographic data, medical history, sun exposure habits, and photoprotection behaviors, this study offers a comprehensive analysis of the factors associated with skin cancer.

The study highlights the significant gaps in photoprotection knowledge among skin cancer patients. With only 37.3% of the participants recalling photoprotection education, there is a clear need for enhanced public health initiatives. These findings provide a basis for future educational campaigns tailored to community needs. The comparative analysis of the photoprotection behaviors among the patients with different skin cancer types—BCC, SCC, and melanoma—offers nuanced insights into the distinct patterns of sun exposure and protective measures. This differentiation could help healthcare providers offer tailored advice based on the specific risks for each type of skin cancer.

The key findings include that less than half of the skin cancer patients reported regular sunscreen use. The adherence to sun-safe behaviors was particularly low among the SCC patients, despite their higher occupational sun exposure levels. This reflects a disconnect between risk perception and protective behaviors in this group. Only one-third of the participants recalled receiving photoprotection education, with most expressing a desire for more information from healthcare professionals. This underscores the need for improved education from trusted medical sources. Mediterranean populations, such as those in Crete, typically have Fitzpatrick skin types IV and V, providing some natural protection against UV radiation. In contrast, populations in northern latitudes, such as Scandinavia, are more vulnerable to UV damage due to their lighter skin types (Fitzpatrick I and II). Studies in Australia and California similarly show the impact of high UV exposure, with Mediterranean-like regions experiencing significant occupational sun exposure (67.5% in Crete) and long sunshine durations, contributing to increased skin cancer rates.

This study has several limitations. The study’s cross-sectional design limits the ability to establish causal relationships between sun exposure, photoprotection behaviors, and skin cancer development. Longitudinal studies would offer clearer insights. Self-reported questionnaires may introduce inaccuracies, particularly for childhood sun exposure and past photoprotection habits. The study’s focus on Crete may limit the generalizability to other regions with different climates or cultural attitudes toward sun protection. A smaller healthy control group compared to the patient group may have affected the statistical power for certain comparisons. The recruitment from a dermatology department may overrepresent more severe or advanced skin cancer cases.

## 5. Conclusions

This study highlights the significant associations between skin cancer risk and factors such as sun exposure, photoprotection behaviors, and demographic characteristics in a Greek cohort. The patients with skin cancer, particularly those with lighter skin phototypes, reported greater occupational and leisure sun exposure as well as inadequate photoprotection compared to the healthy controls. While nearly half of the patients reported using sunscreen, it was often with lower SPF ratings, and other protective measures—such as wearing sunglasses, hats, and long sleeves—were less frequently adopted. These findings underscore the critical need for targeted prevention strategies, including public education on comprehensive photoprotection practices, early intervention during childhood, and the promotion of sun-safe behaviors, particularly among high-risk populations. Future research should aim to expand the study population, incorporate longitudinal data, and evaluate the effectiveness of tailored educational programs in improving sun-safe practices in high-risk groups.

## Figures and Tables

**Table 1:** The demographic and clinical characteristics, along with the sun exposure data, of the 265 skin cancer patients overall, categorized by skin cancer type—basal cell carcinoma (BCC) (n = 134), squamous cell carcinoma (SCC) (n = 93), and malignant melanoma (MM) (n = 38)—and 106 healthy controls with no past medical history (PMH) of skin cancer, totaling 371 participants included in this study.

	Patients with Basal Cell Carcinoma (BCC) n = 134/371 (36.1%)	Patients with Squamous Cell Carcinoma (SCC) n = 93/371 (25.1%)	Patients with Malignant Melanoma (MM) n = 38/371 (10.2%)	Patients with No Skin Cancer (Control Group) n = 106/371 (28.6%)	All Participants, n = 371	All Participants, n = 371 C.I.	*p*-Value	Logistic Regression
Mean/median age (±SD)	73.58/ 73 ±SD 11.10	76.19/ 76 ±SD 9.25	57.45/62 ±SD 10.75	75.73/76 ±SD 9.69	73.20/74 ±SD 11.55	72.02–74.38	*p* = 0.678	*p* = 0.621
Age group							*p* = 0.924	*p* = 0.544
≤35- to <55-year-old	10/134 (7.5%)	0/93 (0%)	14/38 (36.8%)	1/106 (0.9%)	25/371 (6.7%)			
≤55- to <75-year-old	56/134 (41.8%)	39/93 (41.9%)	24/38 (63.2%)	44/106 (41.5%)	163/371 (43.9%)			
≤75-year old	68/134 (50.7%)	54/93 (58.1%)	0/38 (0%)	61/106 (57.5%)	183/371 (49.3%)			
Gender, n (%)							*p* = 0.874	*p* = 0.769
Male	80/134 (59.7%)	54/93 (58.1%)	20/38 (52.6%)	43/106 (40.6%)	217/371 (58.5%)			
Female	54/134 (40.3%)	39/93 (41.9%)	18/38 (47.4%)	63/106 (59.4%)	154/371 (41.5%)			
Employment Status, n (%)							*p* = 0.266	*p* = 0.305
Student	0/134 (0%)	0/93 (0%)	0/38 (0%)	0/106 (0%)	0/371 (0%)			
Employed	56/134 (41.8%)	39/93 (41.9%)	38/38 (100%)	44/106 (41.5%)	177/371 (47.7%)			
Unemployed	0/134 (0%)	0/93 (0%)	0/38 (0%)	0/106 (0%)	0/265 (0%)			
Retired	78/134 (58.2%)	51/93 (54.8%)	0/38 (0%)	60/106 (56.6%)	189/371 (50.9%)			
Housewife		3/93 (3.2%)	0/38 (0%)	2/106 (1.9%)	5/371 (1.3%)			
Educational level, n (%)							*p* = 0.000	*p* = 0.003
Elementary school	62/134 (46.3%)	42/93 (45.2%)	6/38 (15.8%)	16/106 (15.1%)	126/371 (34%)			
High school	40/134 (29.9%)	33/93 (35.5%)	21/38 (55.3%)	39/106 (36.8%)	133/371 (35.8%)			
Technical studies	20/134 (14.9%)	15/93 (16.1%)	2/38 (5.3%)	30/106 (28.3%)	67/371 (18.1%)			
University level	12/134 (9%)	3/93 (3.2%)	9/38 (23.7%)	21/106 (19.8%)	45/371 (12.1%)			
Fitzpatrick skin phototype, n (%)							*p* = 0.001	*p* = 0.011
Skin type I (always burns, does not tan)	4/134 (3%)	12/93 (12.9%)	3/38 (7.9%)	0/106 (0%)	19/371 (5.1%)			
Skin type II (burns easily, tans poorly)	32/134 (23.9%)	30/93 (32.3%)	9/38 (23.7%)	11/106 (10.4%)	82/371 (22.1%)			
Skin type III (tans after initial burn)	74/134 (55.2%)	42/93 (45.2%)	24/38 (63.2%)	65/106 (61.3%)	205/371 (55.3%)			
Skin type IV (burns minimally, tans easily)	24/134 (17.9%)	9/93 (9.7%)	2/38 (5.3%)	30/106 (28.3%)	65/371 (17.5%)			
Physician’s Global Assessment of Disease (PGA)							*p* = 0.000	*p* = 0.000
I	42/134 (31.3%)	24/93 (25.8%)	3/38 (7.9%)	106/106 (100%)	175/371 (47.2%)			
II	36/134 (26.9%)	18/93 (19.4%)	9/38 (23.7%)	0/106 (0%)	69/371 (18.6%)			
III	36/134 (26.9%)	39/93 (41.9%)	24/38 (63.2%)	0/106 (0%)	81/371 (21.8%)			
IV	20/134 (14.9%)	12/93 (12.9%)	2/38 (5.3%)	0/106 (0%)	46/371 (12.4%)			
BMI mean/ median (±SD)	27.48/ 26.81 ±SD 4.52	28.29/27.68 ±SD 4.65	29.67/29.07 ±SD 2.88	28.22/27.61 ±4.59	28.12/27.68 ±SD 4.46	27.66–28.57	*p* = 0.119	*p* = 0.309
BMI category							*p* = 0.015	*p* = 0.037
Normal	54/134 (40.3%)	21/93 (22.6%)	3/38 (7.9%)	25/106 (23.6%)	103/371 (27.8%)			
Overweight	40/134 (29.9%)	36/93 (38.7%)	20/38 (52.6%)	42/106 (39.6%)	138/371 (37.2%)			
Obese	40/134 (29.9%)	36/93 (38.7%)	15/38 (39.5%)	39/106 (36.8%)	130/371 (35%)			
Eye color, n (%)							*p* = 0.020	*p* = 0.583
Dark	24/134 (17.9%)	3/93 (3.2%)	2/38 (5.3%)	5/106 (4.7%)	34/371 (9.2%)			
Brown	76/134 (56.7%)	45/93 (48.4%)	24/38 (63.2%)	100/106 (94.3%)	245/371 (66%)			
Blue	22/134 (16.4%)	27/93 (29%)	6/38 (15.8%)	0/106 (0%)	55/371 (14.8%)			
Green	12/134 (9%)	18/93 (19.4%)	6/38 (15.8%)	1/106 (0.9%)	37/371 (10%)			
Natural hair color, n (%)							*p* = 0.189	*p* = 0.138
Red	0/134 (0%)	3/93 (3.2%)	3/38 (7.9%)	0/106 (0%)	6/371 (1.6%)			
Blond	16/134 (11.9%)	18/93 (19.4%)	3/38 (7.9%)	1/106 (0.9%)	38/371 (10.2%)			
Brown	64/134 (47.8%)	48/93 (51.6%)	29/38 (76.3%)	84/106 (79.2%)	225/371 (60.6%)			
Black	54/134 (40.3%)	24/93 (25.8%)	3/38 (7.9%)	21/106 (19.8%)	102/371 (27.5%)			
Number of nevi, n (%)							*p* = 0.839	*p* = 0.973
<25 nevi	116/134 (86.6%)	81/93 (87.1%)	15/38 (39.5%)	100/106 (94.3%)	312/371 (84.1%)			
25–50 nevi	10/134 (7.5%)	12/93 (12.9%)	9/38 (23.7%)	4/106 (3.8%)	35/371 (9.4%)			
50–100 nevi	4/134 (3%)	0/93 (0%)	3/38 (7.9%)	2/106 (1.9%)	9/371 (2.4%)			
100 nevi	4/134 (3%)	0/93 (0%)	11/38 (28.9%)	0/106 (0%)	15/371 (4%)			
Smoking status, n (%)							*p* = 0.182	*p* = 0.212
Current smoker	46/134 (34.3%)	39/93 (41.9%)	11/38 (28.9%)	8/106 (7.5%)	104/371 (28%)			
Nonsmoker	52/134 (38.8%)	39/93 (41.9%)	12/38 (31.6%)	91/106 (85.5%)	194/371 (52.3%)			
Ex-smoker	36/134 (26.9%)	15/93 (16.1%)	15/38 (39.5%)	7/106 (6.6%)	73/371 (19.7%)			
Sunburn before the age of 18, n (%)							*p* = 0.000	*p* = 0.550
No	92/134 (68.7%)	51/93 (54.8%)	14/38 (36.8%)	99/106 (93.4%)	256/371 (69%)			
Yes	42/134 (31.3%)	42/93 (45.2%)	24/38 (63.2%)	7/106 (6.6%)	115/371 (31%)			
Leisure sun exposure, n (%)							*p* = 0.000	*p* = 0.000
No	84/134 (62.7%)	66/93 (71%)	27/38 (71.1%)	99/106 (93.4%)	276/371 (74.4%)			
Yes	50/134 (37.3%)	27/93 (29%)	11/38 (28.9%)	7/106 (6.6%)	95/371 (25.6%)			
Occupational sun exposure, n (%)							*p* = 0.000	*p* = 0.000
No	42/134 (31.3%)	27/93 (29 %)	20/38 (52.6%)	100/106 (94.3%)	186/371 (50.1%)			
Yes	92/134 (68.7%)	66/93 (71 %)	18/38 (47.4%)	6/106 (5.7%)	185/371 (49.9%)			
Mean/median (±SD) weeks of vacation spent before the age of 10 (±SD)	7.91/8 ±SD 3.89	8.77/8 ±SD 4.26	8/8 ±SD 3.69	2.94/3 ±SD 1.49	6.72/6 ±SD 4.21	C.I. 6.29–7.15	*p* = 0.000	*p* = 0.034
Mean/median (±SD) weeks of vacation spent before from the age of 11 until 18 (±SD)	7.46/6 ±SD 3.99	8.14/8 ±SD 4.37	7.68/8 ±SD 3.49	2.40/2 ±SD 1.03	6.21/4 ±SD 4.23	C.I. 5.78–6.07	*p* = 0.000	*p* = 0.030
Mean/median (±SD) weeks of vacation spent after the age of 18 (±SD)	6.79/5 ±SD 3.71	7.31/8 (±4.9)	6.79/ 8 ±SD 4.67	2.25/2 ±1.69	5.63/4 ±SD 4.317	C.I. 5.19–6.07	*p* = 0.000	*p* = 0.635

**Table 2:** Sun protection practices in the 265 skin cancer patients overall, categorized by skin cancer type—basal cell carcinoma (BCC) (n = 134), squamous cell carcinoma (SCC) (n = 93), and malignant melanoma (MM) (n = 38)—and 106 healthy controls with no past medical history (PMH) of skin cancer, totaling 371 participants included in this study.

	Patients with Basal Cell Carcinoma (BCC) n = 134/265 (50.6%)	Patients with Squamous Cell Carcinoma (SCC) n = 93/265 (35.1%)	Patients with Malignant Melanoma (MM) n = 38/265 (14.3%)	Patients with no Skin Cancer (Control Group) n = 106/371 (28.6%)	All Participants, N = 371	*p*-Value	Logistic Regression
Do you use sunscreen? n, %						*p* = 0.000	*p* = 0.035
No	66/134 (49.3%)	53/93 (57%)	12/38 (31.6%)	27/106 (25.5%)	158/371 (42.6%)		
Yes	68/134 (50.7%)	40/93 (43%)	26/38 (68.4%)	79/106 (74.5%)	213/371 (57.4%)		
If yes, which SPF sunblock rating do you use? n, %						*p* = 0.000	*p* = 0.04
<30	26/134 (19.4%)	13/93 (14%)	5/38 (13.2%)	6/106 (5.7%)	158/371 (42.6%)		
≥30	12/134 (9%)	9/93 (9.7%)	6/38 (15.8%)	17/106 (16%)	50/371 (13.5%)		
≥50	30/134 (22.4%)	18/93 (19.4%)	15/38 (39.5%)	56/106 (52.8%)	44/371 (11.9%)		
No sunscreen use	66/134 (49.3%)	53/93 (57%)	12/38 (31.6%)	27/106 (25.5%)	119/371 (32%)		
How frequently do you use sunscreen? n, %						*p* = 0.003	*p* = 0.015
Every day	8/134 (6%)	13/93 (14%)	11/38 (28.9%)	6/106 (5.7%)	50/371 (13.5%)		
Most days	12/134 (9%)	2/93 (2.2%)	3/38 (7.9%)	17/106 (16%)	44/371 (11.9%)		
Occasionally	32/134 (23.9%)	19/93 (20.4%)	9/38 (23.7%)	56/106 (52.8%)	119/371 (32.1%)		
Rarely	16/134 (11.9%)	6/93 (6.5%)	3/38 (7.9%)	0/106 (0%)	0/371 (0%)		
No sunscreen use	66/134 (49.3%)	53/93 (57%)	12/38 (31.6%)	27/106 (25.5%)	158/371 (42.6%)		
During which seasons do you apply sunscreen? n, %						*p* = 0.077	*p* = 0.22
Only during the summer	56/134 (41.8%)	31/93 (33.3%)	23/38 (60.5%)	59/106 (55.7%)	157/371 (42.3%)		
All year round	12/134 (9%)	9/93 (9.7%)	3/38 (7.9%)	20/371 (18.9%)	56/371 (15.1%)		
No sunscreen use	66/134 (49.3%)	53/93 (57%)	12/38 (31.6%)	27/106 (25.5%)	158/371 (42.6%)		
In which of the following weather conditions do you apply sunscreen? n, %						*p* = 0.000	*p* = 0.142
Only in direct sunny weather	57/134 (42.5%)	37/93 (39.8%)	23/38 (60.5%)	41/106 (38.7%)	158/371 (42.6%)		
In both sunny and cloudy weather	11/134 (8.2%)	3/93 (3.2%)	3/38 (7.9%)	38/106 (35.8%)	55/371 (14.8%)		
No sunscreen use	66/134 (49.3%)	53/93 (57%)	12/38 (31.6%)	27/106 (25.5%)	158/371 (42.6%)		
While outdoors, do you reapply sunscreen? n, %						*p* = 0.00	*p* = 0.001
No	105/134 (78.4%)	84/93 (90.3%)	26/38 (68.4%)	30/106 (28.3%)	243/371 (65.5%)		
Yes	29/134 (29%)	9/93 (9.7%)	12/38 (31.6%)	76/106 (71.7%)	128/371 (34.5%)		
Do you reapply sunscreen after swimming or perspiring heavily? n, %						*p* = 0.300	*p* = 0.204
No	111/134 (82.8%)	79/93 (84.9%)	28/38 (73.7%)	37/106 (34.99%)	254/371 (68.5%)		
Yes	23/134 (17.2%)	14/93 (15.1%)	10/38 (26.3%)	69/106 (65.1%)	117/371 (31.5%)		
Wearing UV-protective sunglasses. n, %						*p* = 0.03	*p* = 0.000
Every day	18/134 (13.4%)	6/93 (6.5%)	9/38 (34.6%)	63/106 (59.4%)	97/371 (26.1%)		
Most days	16/134 (11.9%)	9/93 (9.7%)	8/38 (30.8%)	39/106 (36.8%)	77/371 (20.8%)		
Occasionally	26/134 (19.4%)	18/93 (19.4%)	6/38 (23.1%)	3/106 (2.8%)	53/371 (14.3%)		
Rarely	14/134 (10.4%)	6/93 (6.5%)	0/38 (0%)	0/106 (0%)	20/371 (5.4%)		
Never	60/134 (44.8%)	54/93 (58.1%)	3/38 (11.5%)	1/106 (0.9%)	124/371 (33.4%)		
Wearing a broad-brimmed hat. n, %						*p* = 0.001	*p* = 0.02
Every day	10/134 (7.5%)	9/93 (9.7%)	6/38 (23.1%)	16/106 (15.1%)	41/371 (11.1%)		
Most days	32/134 (23.9%)	9/93 (9.7%)	6/38 (23.1%)	22/106 (20.8%)	69/371 (18.6%)		
Occasionally	32/134 (23.9%)	24/93 (25.8%)	3/38 (11.5%)	41/106 (38.7%)	106/371 (28.6%)		
Rarely	10/134 (7.5%)	9/93 (9.7%)	11/38 (42.3%)	5/106 (4.7%)	35/371 (9.4%)		
Never	50/134 (37.3%)	42/93 (45.2%)	0/38 (0%)	22/106 (20.8%)	120/371 (32.3%)		
Wearing long-sleeved shirts or long plants made from tight fabric weave. n, %						*p* = 0.272	*p* = 0.32
Every day	16/134 (11.9%)	6/93 (6.5%)	3/38 (11.5%)	68/106 (64.2%)	41/371 (11.1%)		
Most days	28/134 (20.9%)	15/93 (16.1%)	6/38 (23.1%)	17/106 (16%)	69/371 (18.6%)		
Occasionally	34/134 (25.4%)	27/93 (29%)	9/38 (34.6%)	11/106 (10.4%)	106/371 (28.6%)		
Rarely	24/134 (17.9%)	9/93 (9.7%)	2/38 (7.7%)	5/106 (4.7%)	35/371 (9.4%)		
Never	32/134 (23.9%)	36/93 (38.7%)	6/38 (23.1%)	5/106 (4.7%)	120/371 (32.3%)		
Avoiding the sun during hours of peak sunlight intensity (10:00 am to 16:00 pm). n, %							
Every day	18/134 (13.4%)	9/93 (9.7%)	8/38 (30.8%)	46/106 (43.4%)	81/371 (21.8%)		
Most days	40/134 (29.9%)	24/93 (25.8%)	9/38 (34.6%)	38/106 (35.8%)	114/371 (30.7%)		
Occasionally	38/134 (28.4%)	18/93 (19.4%)	9/38 (34.6%)	14/106 (13.2%)	82/371 (22.1%)		
Rarely	18/134 (13.4%)	12/93 (12.9%)	0/38 (0%)	3/106 (2.8%)	36/371 (9.7%)		
Never	20/134 (14.9%)	30/93 (32.3%)	0/38 (0%)	5/106 (4.7%)	58/371 (15.6%)		

## Data Availability

The data that support the findings of this study are available upon reasonable request from the corresponding author, D.K., due to privacy restrictions.

## Supplementary Materials

The following supporting information can be downloaded at: https://www.mdpi.com/article/10.3390/cancers16244226/s1, Table S1: Sun protection education in 265 skin cancer patients, categorized by skin cancer type—Basal Cell Carcinoma (BCC) (n = 134), Squamous Cell Carcinoma (SCC) (n = 93), and Malignant Melanoma (MM) (n = 38)—and 106 healthy controls with no past medical history (PMH) of skin cancer, totaling 371 participants included in the study; Table S2: Perceived barriers to the implementation of photoprotection practices in 265 skin cancer patients, categorized by skin cancer type—Basal Cell Carcinoma (BCC) (n = 134), Squamous Cell Carcinoma (SCC) (n = 93), and Malignant Melanoma (MM) (n = 38)—and 106 healthy controls with no past medical history (PMH) of skin cancer, totaling 371 participants included in the study; Questionnaire file.
