# Supplementary material for: Skin Cancer Knowledge, Sun Exposure, Photoprotection Behavior, and Perceived Barriers Associated with Skin Cancer Types in a Greek Cohort: A Cross-Sectional Study on the Island of Crete
Source: Cancers (Basel). 2024 Dec 18;16(24):4226. doi: 10.3390/cancers16244226 (PMC11726760; doi:10.3390/cancers16244226)
Supplement: Supplementary file 1 [file cancers-16-04226-s001.zip › Questionnaire English Version.pdf]

## Mole Clinic

**Date:**
**First Name: Last Name: Father's Name:**
**Date of Birth: Age:**
**Patient Registration Number:**
**Address: Postal Code: Area:**
**Email:**
**Mobile Phone Number: Landline Number:**

1. **Gender:**
   □ Male □ Female

**Do you have a personal history of melanoma?** □ Yes □ No
If yes, what year was the diagnosis made?
If you had melanoma, what year was it removed?
Where on your body was the melanoma located?
When was it removed?
What type of melanoma was it?
□ Superficial spreading melanoma
□ Nodular melanoma
□ Melanoma arising on malignant mole
□ Acral lentiginous melanoma
□ Amelanotic melanoma
□ Desmoplastic melanoma
□ Spitzoid melanoma
□ Small cell melanoma
□ Other type of melanoma, please specify:

2. **Nationality:**
3. **Place of Birth:**
4. **Where do you live?**
   □ City
   □ Rural area
5. **What is your level of education?**
   □ Primary school
   □ High school
   □ Technical studies
   □ University degree
6. **What is/was your profession?**
   Occupation:
   □ Student
   □ Employed
   □ Unemployed
   □ Retired
   □ Homemaker
7. **Where did you live until the age of 15?**
8. **What is your skin type?**
   □ Type I: Very fair skin (always burns, never tans)
   □ Type II: Fair skin (burns easily, tans with difficulty)

□ Type III: Light brown skin (burns lightly, tans moderately)
□ Type IV: Brown skin (burns minimally, tans easily)
□ Type V: Dark brown skin (rarely burns, tans easily)
□ Type VI: Black skin (never burns, always tans)

9. **How does your skin react to sun exposure during the summer?**
   (Describe the color of your skin and how it reacts to the sun)
   □ My skin always burns, never tans
   □ My skin always burns, tans a little or with difficulty
   □ My skin burns and then tans
   □ My skin burns very little, tans easily
   □ My skin rarely burns, tans very easily
   □ My skin never burns, always tans
10. **What is your natural hair color?**
    a) Black b) Brown c) Blonde d) Red e) Other, please specify:
11. **What is your natural eye color?**
    a) Black b) Brown c) Blue d) Green e) Other
12. **Are you currently taking any immunosuppressive medication?** Circle what applies:
    A) Azathioprine
    B) Cyclosporine
    C) Methylprednisolone (Medrol)
    D) Prednisolone
    E) Mycophenolate (Cellcept)
    F) Tacrolimus
    G) Sirolimus
    H) Other medication
13. **Have you ever had an organ transplant?** □ Yes □ No
    If yes, what year did the transplant occur?
14. **What type of organ was transplanted?**
    A) Kidney B) Liver C) Heart D) Lungs E) Pancreas
15. **Do you have inflammatory bowel disease?** □ Yes □ No
16. **Do you have ulcerative colitis?** □ Yes □ No, if yes, when was it diagnosed?
17. **Do you have Crohn's disease?** □ Yes □ No, if yes, when was it diagnosed?
18. **Have you ever suffered a severe sunburn?**
    (A sunburn with pain, significant redness, or blisters lasting 2 or more days before the age of 18)
    □ No
    □ Yes
    □ I don't remember
    □ If yes, how many times have you had severe sunburn before the age of 18?
    □ If yes, how many times have you had severe sunburn after the age of 18?
19. **Do you use sunscreen?** □ Yes □ No
20. **If you use sunscreen, what SPF do you use?**
    □ $<30$ SPF
    □ $\geq 30$ SPF
    □ $\geq 50$ SPF
21. **How often do you use sunscreen?**
    □ Every day
    □ Most days

□ Occasionally
□ Rarely

22. **In which seasons do you use sunscreen?**
    □ Only in the summer
    □ All year round
23. **Under which weather conditions do you apply sunscreen?**
    □ Only when exposed to direct sunlight
    □ In both sunny and cloudy/rainy weather
24. **When you are outdoors, do you reapply sunscreen?** □ Yes □ No
25. **If yes, how often do you reapply sunscreen?**
    □ Every 1 hour □ Every 1-2 hours □ Every 2 hours □ Every 2-3 hours
    □ Every 2-4 hours □ Every 3 hours □ Every 3-4 hours □ Every 4 hours
    □ Every 4-6 hours □ Every 5 hours □ Every 6 hours □ Every 6-8 hours □ Every 8 hours
26. **Do you reapply sunscreen after swimming or intense sweating?**
    □ Yes □ No
27. **How often do you use sunscreen when exposed to the sun?**
    (When outdoors for more than one hour, excluding sunbathing):
    □ Never
    □ Sometimes
    □ Always
28. **When you sunbathe**
    □ Never
    □ Sometimes
    □ Always
    **Do you sunbathe?**
    □ Yes □ No
29. **Do you wear UV-protective sunglasses?**
    □ Every day □ Most days □ Occasionally □ Rarely □ Never
30. **Do you wear a wide-brimmed hat?**
    □ Every day □ Most days □ Occasionally □ Rarely □ Never
31. **Do you wear long-sleeved shirts or long pants made of tight fabric?**
    □ Every day □ Most days □ Occasionally □ Rarely □ Never
32. **Do you avoid the sun during peak sun hours (10:00 AM to 4:00 PM)?**
    □ Every day □ Most days □ Occasionally □ Rarely □ Never
33. **Have you ever received advice on how to protect your skin from the sun's rays?**
    □ Yes □ No
34. **Have you ever received sun protection training from any of the following medical specialties?**
    □ General practitioner □ Yes □ No
    □ Rural doctor □ Yes □ No
    □ Internist □ Yes □ No
    □ Dermatologist □ Yes □ No
    □ Cardiologist □ Yes □ No
    □ Gastroenterologist □ Yes □ No
    □ Pulmonologist □ Yes □ No
    □ Nephrologist □ Yes □ No
    □ Surgeon □ Yes □ No
    □ Other specialty □ Yes □ No, please specify

□ Nursing staff □ Yes □ No
□ TV □ Yes □ No
□ Newspaper □ Yes □ No
□ Magazine □ Yes □ No
□ Other form of information (if yes, specify):

35. **How many times has a healthcare professional offered you advice on sun protection?**
    □ Never □ Once □ Twice □ Three times □ More than three times
36. **When did you receive instructions on sun protection?** Year:
37. **If you had an organ transplant, did you receive instructions on sun protection?**
    A) Before the transplant
    B) Immediately after the transplant
    C) 1 to 6 months after the transplant
    D) One year after the transplant
38. **If you had skin cancer, did you receive instructions on sun protection?**
    A) Before the skin cancer
    B) Immediately after the skin cancer
    C) 1 to 6 months after
    D) One year after
39. **If you are immunosuppressed (please specify), did you receive instructions on sun protection?**
    A) Before immunosuppression
    B) Immediately after
    C) 1 to 6 months after
    D) One year after
40. **If you are taking immunosuppressive treatment, did you receive instructions on sun protection?**
    A) Before the treatment started
    B) Immediately after
    C) 1 to 6 months after
    D) One year after
41. **If you have ulcerative colitis or Crohn's disease, did you receive instructions on sun protection?**
    A) Before the diagnosis
    B) Immediately after the diagnosis
    C) 1 to 6 months after
    D) One year after

**Have you ever received written information about sun protection?**
□ Yes □ No

41. **Would you be interested in receiving education on sun protection through the following methods?**
    A) Written brochure or newsletter about sun protection?
    □ Yes □ No
    B) Verbal advice from a healthcare professional?
    □ Yes □ No
    C) Media-based information (e.g., online webinars)?
    □ Yes □ No

D) Do you think you have received sufficient education on sun protection?
□ Yes □ No

**42. Are any of the following obstacles discouraging you from practicing sun protection and self-examining your skin? (Please check all that apply)**

A) Lack of information ("I didn't know that sun protection is important")
□ Yes □ No

B) Skepticism ("I don't believe skin cancer is a serious health threat")
□ Yes □ No

C) Inconvenience / Lack of time
□ Yes □ No

D) Concerns about sufficient Vitamin D
□ Yes □ No

E) Cost / Financial concerns
□ Yes □ No

F) Appearance ("I don't like how I look when using sun protection measures, such as sunscreen, hats, sunglasses...")
□ Yes □ No

G) Difficulty obtaining materials (sunscreen, sunglasses, hats, etc.)
□ Yes □ No

H) Sunscreen is uncomfortable or unpleasant
□ Yes □ No

I) Previous "bad" reaction to sunscreen (please specify)
□ Yes □ No

J) Nothing / No obstacle discourages me
□ Yes □ No

**If you have a personal history of melanoma, did this diagnosis change your behavior regarding sun protection after the diagnosis of melanoma?**
□ Yes □ No

**Before the melanoma diagnosis, what was your behavior regarding sun protection measures?**

Did you use sunscreen before the melanoma diagnosis?
□ Yes □ No

If you used sunscreen before the melanoma diagnosis, what SPF rating did you use?
□ <30 SPF
□ ≥30 SPF
□ ≥50 SPF

How often did you use sunscreen before the melanoma diagnosis?
□ Every day
□ Most days
□ Occasionally (sometimes)
□ Rarely

What seasons did you use sunscreen before the melanoma diagnosis?
□ Only in summer
□ All year round

In which of the following weather conditions did you apply sunscreen before the melanoma diagnosis?
□ Only when exposed to direct sunlight / and when the weather was sunny
□ Both sunny and cloudy/rainy weather

When outdoors, did you reapply sunscreen before the melanoma diagnosis?
□ Yes □ No

If yes, how often did you reapply sunscreen before the melanoma diagnosis?
□ Every 1 hour
□ Every 1-2 hours
□ Every 2 hours
□ Every 2-3 hours
□ Every 2-4 hours
□ Every 3 hours
□ Every 3-4 hours
□ Every 4 hours
□ Every 4-6 hours
□ Every 5 hours
□ Every 6 hours
□ Every 6-8 hours
□ Every 8 hours

Did you reapply sunscreen after swimming or intense sweating before the melanoma diagnosis?
□ Yes □ No

**How often did you use sunscreen when exposed to the sun before the melanoma diagnosis?**

When I was outdoors for more than one hour (other than sunbathing):
□ Never
□ Sometimes
□ Always

When I was sunbathing:
□ Never
□ Sometimes
□ Always

Did you ever sunbathe before the melanoma diagnosis?
□ Yes □ No

When you used sunscreen before the melanoma diagnosis, did you apply it:
□ On your face and body, or
□ Only on your face?

Did you use UV-protective sunglasses before the melanoma diagnosis?
□ Every day
□ Most days
□ Occasionally
□ Rarely
□ Never

Did you wear a wide-brimmed hat before the melanoma diagnosis?
□ Every day
□ Most days
□ Occasionally
□ Rarely
□ Never

Did you avoid the sun during peak sun intensity hours before the melanoma diagnosis? (10:00 AM to 4:00 PM)
□ Every day
□ Most days
□ Occasionally
□ Rarely
□ Never

Were you ever given advice on how to protect your skin from sunlight before the melanoma diagnosis?
□ Yes □ No

---

**43. Did you ever live for one year or more in a country with more intense sunlight than the country you currently live in?**

□ No
□ Yes, before the age of 18: ______ years
□ Yes, after the age of 18: ______ years

**44. SUN EXPOSURE DURING ADULTHOOD:**

**45. Did you work or do you work outdoors? If yes, for how many years?**
□ No
□ Yes, for 1 year or less
□ Yes, for 2-5 years
□ Yes, for 6-10 years
□ Yes, for > 10 years
□ If yes, please specify your job:
□ How many hours per day:
□ How many days per month:
□ How many months per year:
□ How many years:

**46.1 SUN EXPOSURE FOR RECREATION (e.g. outdoor hobbies or physical activities, excluding sunbathing, with at least 4 hours of exposure per day):**
□ No / Yes
□ If yes, specify the activity:
□ How many hours per day:
□ How many days per month:
□ How many months per year:
□ How many years:

**46.2 INTERMITTENT SUN EXPOSURE (e.g., exposure to the sun in spring or summer, including holidays, during peak hours from 11 AM to 4 PM)**
Age | Exposure time | Weeks of vacation | Hours of sun exposure from 11 AM to 4 PM
Childhood (up to 10 years)
Adolescence (11-18 years)
Adulthood (>18 years)
Last 10 years for healthy individuals or
Last 10 years before melanoma diagnosis (for melanoma patients)

**46.3 Most recent intense intermittent sun exposure:**
Please specify the date or month of the most recent intense intermittent sun exposure before the melanoma diagnosis (for patients) or before this interview (for healthy individuals).

**46.4 Did you ever have a severe or painful sunburn before the age of 18?**
□ Yes □ No

**46.5 Have you spent significant time outdoors for work or recreation?**
□ Yes □ No

**46.6 Have you ever been diagnosed with skin cancer?**
□ Yes □ No
If yes, what year and type of cancer?
A) Basal cell carcinoma
B) Squamous cell carcinoma
C) Melanoma
D) I don’t know
E) Other

**46.7 Has any close relative been diagnosed with skin cancer? (e.g., parents, siblings, children)**
□ Yes □ No, and who?

**46.8 Severe burns (second-degree) during your life:**
Definition: Burn with redness and pain for more than two days or with blisters (duration doesn't matter)
□ Before the age of 18, Yes / No / I don't know
If yes, number:
□ After the age of 18, Yes / No / I don't know
If yes, number:
□ In the melanoma area? Yes / No / I don't know
If yes, number:

□ In the last five years: Yes / No / I don't know
If yes, number:

---

**47.1 Number of weeks per year on holidays in sunny places:**
□ 0 weeks per year
□ 2 weeks per year or less
□ More than 2 weeks per year

**47.2 Do you expose yourself to the sun in order to tan?**
□ Yes □ No

**47.3 Do you use artificial tanning (solarium)?**
□ No
□ Yes, less than 20 times per year
□ Yes, 21 or more times per year

**47.4 How many years have you been using artificial tanning (solarium)?**
______ years

**47.5 Do you believe that sun exposure for tanning, if you wear sunscreen, is not harmful to the skin?**
□ Yes □ No

**47.6 The purpose of using sunscreen is to avoid sunburn but still be able to tan.**
□ Yes □ No

**47.7 Do you consider tanning with solarium as a protective mechanism before starting sunbathing?**
□ Yes □ No

**47.8 Do you regularly self-examine your skin for skin cancer?**
□ Yes □ No
If yes, how often?
□ Every month

□ Every 3 months
□ Once a year
□ Every 2 years
□ Other frequency, specify:

---

**Physician's global assessment score:** 0, I, II, III, IV, V
**Phototype of skin:** I, II, III, IV, V

---

**EVALUATION OF THE PATIENT**
Evaluation of dermatological problem severity by the patient:
a) Mild
b) Moderate
c) Severe
d) Very severe
0 1 2 3 4 5 6 7 8 9 10

How much pain do you feel due to your dermatological problem?
0 1 2 3 4 5 6 7 8 9 10

How much itching do you feel due to your dermatological problem?
0 1 2 3 4 5 6 7 8 9 10

---

**Skin lesions locations:**
E.g., □ Head □ Face □ Scalp □ Neck □ Trunk □ Back □ Chest □ Upper limbs □ Hands □ Forearms □ Arms □ Lower limbs □ Thighs □ Shins □ Feet

**48. Number of moles during adolescence:**
**49. Number of freckles on the face during adolescence:**

50. WHAT ARE YOUR HOBBIES?
51. WHAT OTHER HEALTH PROBLEMS DO YOU HAVE?
    52.1 DO YOU HAVE A HISTORY OF PROSTATE CANCER?
    □ No
    □ Yes

52.2 DO YOU HAVE A HISTORY OF ENDOMETRIOSIS?
□ No
□ Yes

52.3 HAVE YOU HAD ANY OTHER SKIN CONDITIONS?
(For example: dermatitis, psoriasis)
□ No
□ Yes
If yes, which one?

53. HAVE YOU EVER UNDERGONE PHOTOTHERAPY IN THE PAST?
    53.1 PUVA:
    □ No
    □ Yes
    □ Number of sessions and when:

53.2 UVB:
□ No
□ Yes
□ Number of sessions and when:

54. HISTORY OF PHOTOSENSITIVITY?
    □ Yes
    □ No
55. HAVE YOU EVER RECEIVED TREATMENT IN THE PAST THAT SUPPRESSED YOUR IMMUNE SYSTEM?
    Medication name:
    Dose:
    Duration of immunosuppressive treatment, from: __ to: __

Other medications associated with photosensitivity or immunosuppression:

56. WHAT MEDICATIONS DO YOU TAKE SYSTEMATICALLY?
57. HAVE YOU EVER RECEIVED BIOLOGICAL AGENTS?
    □ No
    □ Yes
    □ From __ to __
58. FAMILY HISTORY OF MELANOMA:
    (Melanoma in first-degree relatives: father, mother, brother, sister)
    □ No
    □ Yes: 1 first-degree relative
    □ Yes: more than 2 first-degree relatives
    □ The patient does not know
59. PERSONAL HISTORY OF SKIN CANCER:
    □ No
    □ Yes, melanoma, year of diagnosis: __, type of melanoma: __
    □ Yes, non-melanoma skin cancer, year of diagnosis: __
    □ The patient does not know
60. NATURAL HAIR COLOR AT THE AGE OF 20:
    □ Red
    □ Blonde
    □ Brown (light brown)
    □ Dark color
61. EYE COLOR:
    □ Dark eye color
    □ Brown
    □ Blue
    □ Green

**Personal medical history:**

- **Rheumatic diseases:** Psoriatic arthritis, Rheumatoid arthritis, Osteoarthritis
- **Joint pain:** Mono-, oligo-, poly-, small joints, large joints
- **Thyroid disease:**
- **Diabetes:**
  Type: __ Duration: __
- **Dyslipidemia:**
  Duration: __ Treatment: __

**Personal history of other dermatological diseases:**

- Personal history of hidradenitis suppurativa, folliculitis, pilonidal cyst, psoriasis
- Personal history of atopy (dermatitis, asthma, allergic rhinitis)

**Psychiatric history:**

**Other medications you are taking:**
Type: __ From: __ To: __
Type: __ From: __ To: __

**Allergies:**

**Weight:** __
**Height:** __
**BMI:** __

**Smoker:**
□ Yes □ No □ Ex-smoker (packs/year: __)
