# Supplementary material for: Skin Cancer Knowledge, Sun Exposure, Photoprotection Behavior, and Perceived Barriers Associated with Skin Cancer Types in a Greek Cohort: A Cross-Sectional Study on the Island of Crete
Source: Cancers (Basel). 2024 Dec 18;16(24):4226. doi: 10.3390/cancers16244226 (PMC11726760; doi:10.3390/cancers16244226)
Supplement: Supplementary file 1 [file cancers-16-04226-s001.zip › Supplementary Table S1.pdf]

Supplementary Table S1: Sun protection education in 265 skin cancer patients, categorized by skin cancer type—Basal Cell Carcinoma (BCC) (n=134), Squamous Cell Carcinoma (SCC) (n=93), and Malignant Melanoma (MM) (n=38)—and 106 healthy controls with no past medical history (PMH) of skin cancer, totaling 371 participants included in the study.

	**Patients with Basal cell carcinoma (BCC) N=134/371 (36.1%)**	**Patients with Squamous cell carcinoma (SCC) N=93/371 (25.1%)**	**Patients with malignant melanoma (MM) N=38/371 (10.2%)**	**Patients with no skin cancer (control group) N=106/371 (28.6%)**	**All participants, N=371**	**p-value**	**Logistic regression**
**Have you ever been given advice on how to protect your skin from sunlight?**						P=0.000	P=0.828
No	88/134 (65.7%)	66/93 (71%)	11/38 (28.9%)	57/106 (53.8%)	215/371 (58%)		
Yes	46/134 (34.3%)	27/93 (29%)	27/38 (71.1%)	49/106 (46.2%)	156/371 (42%)		
**Have you ever received sun protection education from a specialist doctor? N, %**						P=0.000	P=0.097
No sun protection advice has been given, N. %	92/134 (68.7%)	63/93 (67.7%)	11/38 (28.9%)	61/106 (57.5%)	227/371 (61.2%)		
Yes, sun protection advice has been given by a dermatologist, N, %	34/134 (25.4%)	27/93 (29%)	25/38 (65.8%)	41/106 (38.7%)	127/371 (34.2%)		
Yes, sun protection advice has been given by a family doctor, N, %	8/134 (6%)	3/93 (3.2%)	2/38 (5.3%)	4/106 (3.8%)	17/371 (4.6%)		
**On how many occasions have you received sun protection education from a healthcare**						P=0.000	P=0.030

**professional? N, %**							
Never	90/134 (67.2%)	54/93 (58.1%)	9/38 (23.7%)	58/106 (54.7%)	194/371 (52.3%)		
Once	26/134 (19.4%)	18/93 (19.4%)	8/38 (21.1%)	27/106 (25.5%)	78/371 (21%)		
Twice	8/134 (6%)	12/93 (12.9%)	12/38 (31.6%)	12/106 (11.3%)	50/371 (13.5%)		
3 times	4/134 (3%)	3/93 (3.2%)	3/38 (7.9%)	4/106 (3.8%)	17/371 (4.6%)		
>3 times	6/134 (4.5%)	6/93 (6.5%)	6/38 (15.8%)	5/106 (4.7%)	32/371 (8.6%)		
**Have you ever received sun protection education from Media (ie, television, newspaper)? N, %**						P=0.000	P=0.153
No	106/134 (79.1%)	87/93 (93.5%)	21/38 (55.3%)	99/106 (93.4%)	313/371 (84.4 %)		
Yes	28/134 (20.9%)	6/93 (6.5%)	17/38 (44.7%)	7/106 (6.6%)	58/371 (15.6%)		
**Have you ever received written advice about sun protection? N, %**						P=0.014	P=0.215
No	110/134 (82.1%)	87/93 (93.5%)	29/38 (76.3%)	72/106 (67.9%)	298/371 (80.3%)		
Yes	24/134 (17.9%)	6/93 (6.5%)	9/38 (23.7%)	34/106 (32%)	73/371 (19.7%)		
**Would you be interested in receiving education about sun protection? N, %**						P=0.000	P=0.042
No	34/134 (25.4%)	21/93 (22.6%)	0/38 (0%)	5/106 (8.5%)	64/371 (17.3%)		
Yes	100/134 (74.6%)	72/93 (77.4%)	38/38 (100%)	97/106 (91.5%)	307/371 (82.7 %)		
**Would you be interested in receiving photoprotection advice about sun protection from a healthcare worker? N, %**						P=0.008	P=0.027

No	50/134 (37.3%)	21/93 (22.6%)	6/38 (15.8%)	35/106 (33%)	112/371 (30.2%)		
Yes	84/134 (62.7%)	72/93 (77.4%)	32/38 (84.2%)	71/106 (67%)	259/371 (69.8%)		
**Would you be interested in receiving photoprotection advice about sun protection from multimedia? N, %**						P=0.000	P=0.205
No	58/134 (43.3%)	27/93 (29%)	3/38 (7.9%)	32/106 (30.2%)	120/371 (32.3%)		
Yes	76/134 (56.7%)	66/93 (71%)	35/38 (92.1%)	74/106 (69.8%)	251/371 (67.7%)		
