# Supplementary material for: Skin Cancer Knowledge, Sun Exposure, Photoprotection Behavior, and Perceived Barriers Associated with Skin Cancer Types in a Greek Cohort: A Cross-Sectional Study on the Island of Crete
Source: Cancers (Basel). 2024 Dec 18;16(24):4226. doi: 10.3390/cancers16244226 (PMC11726760; doi:10.3390/cancers16244226)
Supplement: Supplementary file 1 [file cancers-16-04226-s001.zip › Supplementary Table S2.pdf]

Supplementary Table S2: Perceived barriers to the implementation of photoprotection practices in 265 skin cancer patients, categorized by skin cancer type—Basal Cell Carcinoma (BCC) (n=134), Squamous Cell Carcinoma (SCC) (n=93), and Malignant Melanoma (MM) (n=38)—and 106 healthy controls with no past medical history (PMH) of skin cancer, totaling 371 participants included in the study.

	**Patients with Basal cell carcinoma (BCC) N=134/371 (36.1%)**	**Patients with Squamous cell carcinoma (SCC) N=93/371 (25.1%)**	**Patients with malignant melanoma (MM) N=38/371 (10.2%)**	**Patients with no skin cancer (control group) N=106/371 (28.6%)**	**All participants, N=371**	**p-value**	**Logistic regression**
**Have any of the following barriers discouraged you from practicing sun protection? N, %**						P=0.566	P=0.192
No	56/134 (41.8%)	45/93 (48.4%)	17/38 (44.7%)	49/106 (46.2%)	167/371 (45%)		
Yes	78/134 (58.2%)	48/93 (51.6%)	21/38 (55.3%)	57/106 (53.8%)	204/371 (55%)		
**Skepticism ("I do not believe skin cancer is a serious health threat"). N, %**						0.842	0.935
No	90/134 (67.2%)	60/93 (64.5%)	29/38 (76.3%)	68/106 (64.2%)	247/371 (66.6%)		
Yes	44/134 (32.8%)	33/93 (35.5%)	9/38 (23.7%)	38/106 (35.8%)	124/371 (33.4%)		
**Hassle/lack of time, N, %**						0.750	0.137
No	96/134 (71.6%)	81/93 (87.1%)	26/38 (68.4%)	81/106 (76.4%)	284/371 (76.5%)		
Yes	38/134 (28.4%)	12/93 (12.9%)	12/38 (31.6%)	25/106 (23.6%)	87/371 (23.5%)		
**Concerns over adequate Vitamin D. N, %**						0.390	0.360
No	106/134 (79.1%)	81/93 (87.1%)	26/38 (68.4%)	82/106 (77.4%)	295/371		

					(79.5%)		
Yes	28/134 (20.9%)	12/93 (12.9%)	12/38 (31.6%)	24/106 (22.6%)	76/371 (20.51%)		
**Cost/financial concerns. N, %**						0.376	0.270
No	94/134 (70.1%)	63/93 (67.7%)	32/38 (84.2%)	66/106 (62.3%)	255/371 (68.7%)		
Yes	40/134 (29.9%)	30/93 (32.3%)	6/38 (15.8%)	40/106 (37.7%)	116/371 (31.3%)		
**Appearance ("I do not like how using sun protection will make me look"). N, %**						0.099	0.147
No	100/134 (74.6%)	72/93 (77.4%)	29/38 (76.3%)	69/106 (65%)	270/371 (72.8%)		
Yes	34/134 (25.4%)	21/93 (22.6%)	9/38 (23.7%)	37/106 (34.9%.1)	101/371 (27.2%)		
**Difficulty obtaining materials (sunscreen, sunglasses, hats, etc). N, %**						0.036	0.04
No	110/134 (82.1%)	78/93 (83.9%)	35/38 (92.1%)	96/106 (90.6%)	319/371 (86%)		
Yes	24/134 (17.9%)	15/93 (16.1%)	3/38 (7.9%)	10/106 (9.4%)	52/371 (14%)		
**Sunscreen is uncomfortable or unpleasant. N, %**						0.456	0.116
No	108/134 (80.6%)	69/93 (74.2%)	23/38 (60.5%)	83/106 (78.3%)	283/371 (76.3%)		
Yes	26/134 (19.4%)	24/93 (25.8%)	15/38 (39.5%)	23/106 (21.7%)	88/371 (23.7%)		
**Previous "bad" reaction to sunscreen (please specify). N, %**							
No	126/134 (94%)	87/93 (93.5%)	38/38 (100%)	100/106 (94.3%)	351/371 (94.6%)		
Yes	8/134 (6%)	6/93 (6.5%)	0/38 (0%)	6/106 (5.7%)	20/371 (5.4%)		
**None/no barriers have discouraged me. N, %**						0.302	0.762

No	114/134 (85.1%)	78/93 (83.9%)	26/38 (68.4%)	87/106 (94.3%)	305/371 (82.2%)
Yes	20/134 (14.9%)	15/93 (16.1%)	12/38 (31.6%)	19/106 (17.9%)	66/371 (17.8%)
